# Characterization of high-molecular weight by-products in the production of a trivalent bispecific 2+1 heterodimeric antibody

**DOI:** 10.1080/19420862.2023.2175312

**Published:** 2023-02-17

**Authors:** Dario A. T. Cramer, Vojtech Franc, Anna-Katharina Heidenreich, Michaela Hook, Mahdi Adibzadeh, Dietmar Reusch, Albert J. R. Heck, Markus Haberger

**Affiliations:** a Biomolecular Mass Spectrometry and Proteomics, Bijvoet Center for Biomolecular Research and Utrecht Institute for Pharmaceutical Sciences, University of Utrecht, Utrecht, The Netherlands; b Netherlands Proteomics Center, Utrecht, The Netherlands; c Pharma Technical Development, Roche Diagnostics GmbH, Penzberg, Germany; d Pharma Technical Development, F. Hoffmann-La Roche AG, Basel, Switzerland

**Keywords:** Native MS, bispecific antibodies, mass photometry, HMW by-products

## Abstract

The development of increasingly complex antibody formats, such as bispecifics, can lead to the formation of increasingly complex high- and low-molecular-weight by-products. Here, we focus on the characterization of high molecular weight species (HMWs) representing the highest complexity of size variants. Standard methods used for product release, such as size exclusion chromatography (SEC), can separate HMW by-products from the main product, but cannot distinguish smaller changes in mass. Here, for the identification of the diverse and complex HMW variants of a trivalent bispecific CrossMAb antibody, offline fractionation, as well as production of HMW by-products combined with comprehensive analytical testing, was applied. Furthermore, HMW variants were analyzed regarding their chemical binding nature and tested in functional assays regarding changes in potency of the variants. Changes in potency were explained by detailed characterization using mass photometry, SDS-PAGE analysis, native mass spectrometry (MS) coupled to SEC and bottom-up proteomics. We identified a major portion of the HMW by-products to be non-covalently linked, leading to dissociation and changes in activity. We also identified and localized high heterogeneity of a by-product of concern and applied a CD3 affinity column coupled to native MS to annotate unexpected by-products. We present here a multi-method approach for the characterization of complex HMW by-products. A better understanding of these by-products is beneficial to guide analytical method development and proper specification setting for therapeutic bispecific antibodies to ensure constant efficacy and patient safety of the product through the assessment of by-products.

## Introduction

Monoclonal antibodies (mAbs) are used extensively as therapeutics in the treatment of various diseases,^1^ with currently around 150 mAbs approved for clinical use.^2^ Beyond the success story of conventional mAbs, structurally more complex bispecific antibodies (bsAbs) offer the possibility of new and tailored modes of action. Compared to standard mAbs, bsAbs target two different antigens simultaneously. Their bi-specificity makes them highly interesting for cancer therapies, to direct and activate immune cells directly at the tumor site.^3,4^ Consequently, these bsAbs can be more potent than conventional antibodies.^5^ Due to this high potential, by now nearly 60 bsAbs are evaluated in clinical trials. With six bsAbs already marketed,^2^ these numbers are expected to increase substantially in the near future.^4,5^

A wide range of technologies is used within the pharmaceutical industry to efficiently produce bsAb formats.^6–8^ Production of these next-generation mAb-based formats is intrinsically more complex than standard mAbs and sometimes leads to unwanted by-products. Size exclusion chromatography (SEC) is routinely used to support process development and release testing^9^ of therapeutics with respect to size variants of the product. In combination with multi-angle light scattering (MALS) detection, absolute masses of the respective ultraviolet (UV) signals can be determined. However, in the case of bsAbs, the resolution of masses is too low for an unambiguous assignment of protein variants. By-products can be resolved with analytical and preparative SEC, but due to its low resolution, SEC cannot resolve and characterize smaller mass deviations. A prominent example of such a complex mAb format is a trivalent bispecific CrossMAb antibody.^10,11^ By-products are expected to form during production as aggregates or as a tetravalent variant. These HMW by-products raise concerns because they may lead to undesired immunogenic responses or differences in potency when present in the final product, as also seen with standard mAbs.^12^

Ideally, unwanted HMW by-products should therefore be removed or kept to acceptable minimal levels to ensure efficacy and safety.^13^ Regardless, it is impossible to completely remove these unwanted by-products in the final formulation. Additionally, HMW by-products like aggregates can potentially form during real-time storage, even at 5°C. Thus, the process development and product understanding must characterize by-products at a molecular level when assessing new antibody formats. Equally important is analyzing and understanding their potency and possible modes of action to determine tolerable levels of by-products in the final product. Finally, a deeper understanding of the formation of by-products may provide clues for future protein design to prevent the formation of by-products.

Mass spectrometry (MS) already plays a pivotal role in characterizing mAbs.^14^ Native MS has matured into a robust and reliable method for the accurate determination of masses of various mAb products,^15–19^ including their by-products. Native MS provides an agnostic view of the protein, with insight into covalent or even non-covalent complex formation^20^ and accurate mass annotation. As a potential bonus, post-translational modifications (PTMs) and structural features can be characterized and monitored by native MS as well, including those that induce small mass shifts such as methionine oxidation.^21^ Additionally, glycosylation profiles and other structural microheterogeneity,^15,22^ aggregates,^20^ charge variants and other PTMs^23^ can be sampled. Similar to SEC coupled to native MS (SEC-nMS), online separation using cationic exchange chromatography (CEC) coupled with native MS (CEC-UV-MS) has been successfully used to analyze antibody product charge variants.^24,25^ Cation CEC-UV-MS was successfully adopted for bsAbs revealing antigen-binding fragment (Fab) glycosylation, including non-consensus N-glycosylation and deamidation.^23^ Variations of native MS have also already been used to detect HMW products of standard antibodies.^26^ However, native MS can *a priori* not characterize all protein properties, such as the nature of unknown modifications and residue-specific information. Here, bottom-up MS like tryptic liquid-chromatography LC-MS/MS peptide mapping is an established method for identifying and quantifying PTMs^27^ and non-reducing SDS-PAGE can provide insight into aggregate formation.^12^ Other techniques, such as mass photometry, a diffraction technique enabling mass determination under near-native conditions, can provide additional insight into chemical binding nature.^28,29^

Here, we present the characterization of a 2 + 1 trivalent bsAb heterodimer (2 + 1 CrossMAb) and all its co-occurring HMW by-products using a combination of techniques. The 2 + 1 CrossMAb and its HMW by-products were fractionated by SE-HPLC and a tetravalent variant was recombinantly expressed as a control. We first compared the functional activity of all isolated HMW by-products to the trivalent product and tetravalent control. Based on the outcome of the bioactivity assessment, we focused on the species showing increased activity compared to the desired product. We used online-coupled SE-HPLC-nMS to assign accurate masses to all the by-products as well as elucidate their chemical binding nature in combination with non-reducing SDS-PAGE and mass photometry. Native MS, bottom-up MS and a CD3 affinity column were applied to annotate expected and unexpected by-products and to reveal their proteoform heterogeneity. By combining all these different analytical techniques, we were able to elucidate all main HMW by-products and assess the risk associated with their presence with respect to the in-vitro activity.

## Results

**Figure 1.**
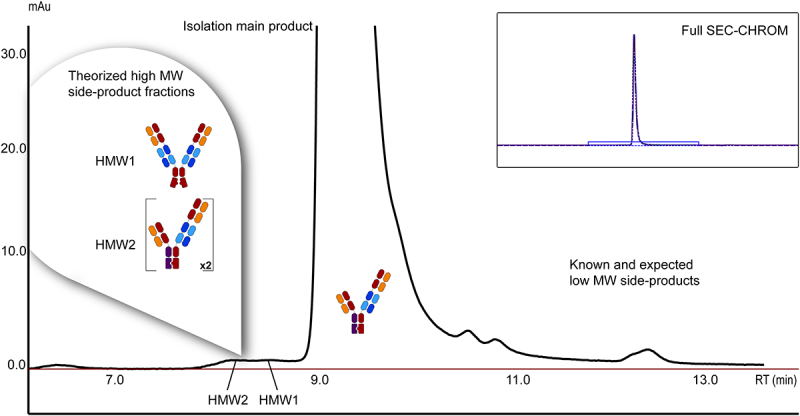
SE-HPLC chromatogram of a Complex Therapeutic 2 + 1 bsAb. During SE-HPLC of the 2 + 1 CrossMAb after protein A purification, HMW by-products were observed at low abundancy, representing less than 1% of the total area under the curve (AUC). The hypothesized by-products are indicated on the peaks representing HMW variants. Indicated fractions were collected for further analysis.

We aimed to characterize the HMW by-products of a 2 + 1 trivalent bsAb heterodimer (2 + 1 CrossMAb) therapeutic product with two identical tumor-cell-specific targeting Fabs and one CD3-targeting Fab. This specific antibody uses two advanced antibody production techniques. CrossMAb^10^ inverts the CD3 Fab light chain (LC), ensuring LCs bind to the corresponding heavy chain (HC) Fab regions. The knob-in-hole technique^30^ homes the bivalent elongated HC to the monovalent HC, forming a disulfide bond and preventing the formation of a tetravalent variant that could crosslink T cells. The final product is designed to hold monovalent specificity for a T cell and a bivalent specificity for an unspecified antigen, inducing T-cell-mediated killing. The molecule selected for this study was produced recombinantly in a Chinese hamster ovary (CHO) cell line in a process identical to standard therapeutic mAb production. SEC analysis of the purified 2 + 1 CrossMAb product revealed that two HMW peaks are present at an abundance below 1.0 area% (Figure 1). Based on the structure of the 2 + 1 CrossMAb, it was hypothesized that two HMW by-products form, a dimer of 396 kDa and/or a tetravalent variant of 245 kDa (Supplementary Figure 1). We pursued the characterization of HMW by-products for critical quality assessment. We fractionated two HMW fractions with preparative SEC after protein A purification of the fermentation broth.

### Potency assays of HMW by-Products

To assess the bioactivity of the HMW by-products compared to the 2 + 1 CrossMAb product, we performed a cell-based reporter gene assay. The activation depended on the simultaneous binding of the Fab region to its target antigen coated on a plate. We aimed to dose-dependently detect the activation of a reporter T cell line. We hypothesized the tetravalent variant (in the HMW1 fraction) to be more potent due to increased T-cell activation. For this reason, we also designed and expressed a tetravalent variant as a positive control. Additionally, we expected the aggregate in fraction HMW2 to have increased potency, as also often seen in mAb aggregates.

**Figure 2.**
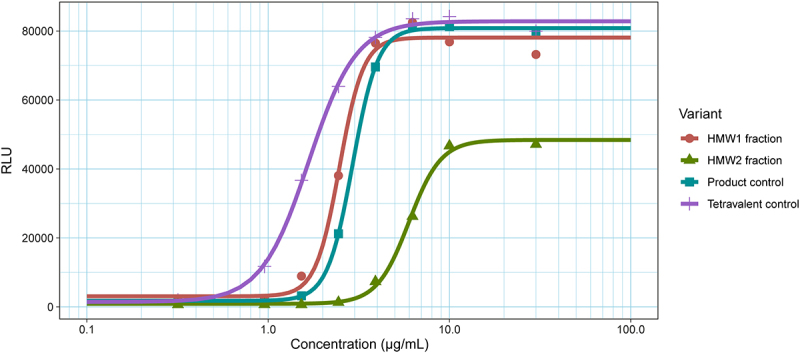
Gene reporter assays measuring variant potency. Dose-response curves show the binding of CD3 Fabs to CD3e expressed on JURKAT cells, meant to simulate downstream signaling. The tetravalent control has the highest potency compared to the 2 + 1 CrossMAb control. The HMW1 fraction, thought to contain the tetravalent variant, shows a remarkably similar response as the product control. The dimer (HMW2 fraction) shows a substantially reduced potency.

Dose-response curves showed an increased potency of the tetravalent control relative to the 2 + 1 CrossMAb (Figure 2). This increased potency revealed its undesired biological activity, which could lead to T-cell auto-activation. Surprisingly, the dose-response curve of the HMW1 fraction revealed similar activity compared to the product control, while we hypothesized it to contain the tetravalent by-product. Also, unexpectedly, the HMW2 fraction containing the dimer showed significantly reduced activity compared to the product control. A possible explanation is that the size of the CrossMAb aggregate prevents similar biological activity due to buried sites at the dimer interface. Based on the observed activities, we identified the dimer as a by-product of lesser concern and focused on the HMW1 fraction. As we identified the tetravalent variant as a by-product of major concern, we performed a potency assay spiking in the separately produced tetravalent control at different concentrations (Supplementary Table 1). Yet, the presence of up to 10% of the tetravalent variant with the 2 + 1 CrossMAb product had no large effect on the bioactivity. This provides a benchmark of safe concentrations of the tetravalent variant, but also shows that the HMW1 fraction contains less tetravalent variant than expected, indicating that other by-products should be present in this fraction.

### SDS-PAGE and mass photometry

**Figure 3.**
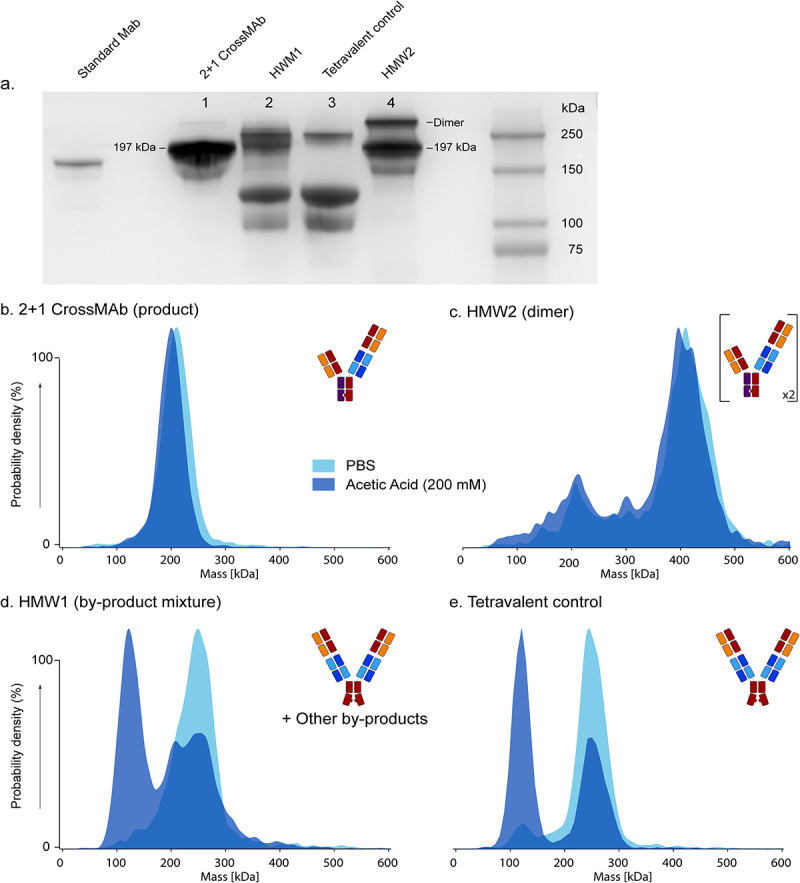
Non-Reducing SDS-PAGE and Mass Photometry Reveal Additional By-products and Non-covalent Interactions in HMW1 and HMW2 Fractions. a. Non-reducing SDS-PAGE analysis reveals the presence of multiple species in the HMW1 fraction and hints at non-covalent binding in the HMW1 fraction and tetravalent control. b-e. Mass photometry under native and acidic conditions of fractions corresponding to the gel lanes in a. Differences in the data between PBS and acidic conditions hint at the presence of non-covalent assemblies.

Based on the potency assays, we expected the HMW1 fraction to contain unexpected by-products. To confirm this hypothesis, we investigated aliquots of the 2 + 1 CrossMAb, HMW1 and HMW2 fractions and tetravalent control by SDS-PAGE under non-reducing conditions (Figure 3a). As a molecular weight marker, we included a standard commercial mAb of ~150 kDa. The 2 + 1 CrossMAb product, with a theoretical mass of 197 kDa, was observed at around 200 kDa. A band estimated to be around 400 kDa was annotated as the dimer, possibly formed in real-time storage after SEC separation.

On SDS-PAGE, the HMW1 fraction showed two distinct bands (Figure 3a) around 250 kDa, revealing that more species are present. The tetravalent control showed a single band around 250 kDa, assigned as the tetravalent variant. Other bands for both the HMW1 fraction and the tetravalent control appear at around 100 kDa, 120 kDa and for the HMW1 fraction at 150 kDa. We interpret the 100 and 120 kDa bands as dissociation products of non-covalent assemblies due to the instability of the tetravalent variant. The 120 kDa band could represent a bivalent knob half-body, half the mass of the tetravalent variant. This suggests the tetravalent variant to be at least partially non-covalently bound. To explain the 100 kDa band, we propose that the knob half-body may have lost a light chain. Such loss of light chains is sometimes also observed in standard MAbs. This also explains the faint band at 150 kDa in HMW1, which matches the mass of a 2 + 1 CrossMAb that has lost two light chains (44–46 kDa). These data imply that the by-products in the HMW1 fraction are not only tetravalent variants.

To corroborate the findings from the SDS-PAGE analysis, we performed mass photometry (Figure 3b-e), which provides information about the mass of single particles by using light diffraction. Aliquots of the 2 + 1 CrossMAb product, the HMW1 and HMW2 fractions and the tetravalent control were measured both under near-native conditions (phosphate-buffered saline (PBS) buffer) and in an acidic environment (pH 2.7 acetic acid, 200 mM). In PBS, the main 2 + 1 CrossMAb product revealed a single peak of ~200 kDa, close to the expected mass of 197 kDa. Bringing this sample to pH 2.7 did not affect the sample (Figure 3b). The HMW2 product displayed a dominant peak around 400 kDa, with a minor peak near 200 kDa. These assemblies also did not dissociate at low pH (Figure 3c). We annotate the larger species as the dimer (theoretical mass 394 kDa) and confirm that the 2 + 1 CrossMAb is present in the HMW2 fraction. The dimer stability at a lower pH suggests it is at least partially covalently bound or stable under denaturing conditions. Mass photometry on the HMW1 fraction revealed a broader mass histogram of species around 250 kDa (Figure 3d). At low pH, we observed the prominent formation of the 120 kDa bivalent half-body in the HMW1 and tetravalent control (Figure 3d-e). The resolution of mass photometry is too low to distinguish multiple species of similar mass around 250 kDa. However, acidification shows that at least a large portion of the HMW1 fraction, as well as the tetravalent control, are non-covalently linked. Thus, the mass photometry data confirmed the presence of additional by-products in the HMW1 fraction and revealed the non-covalent binding nature of the tetravalent variant.

### Characterization by SEC-nMS

**Figure 4.**
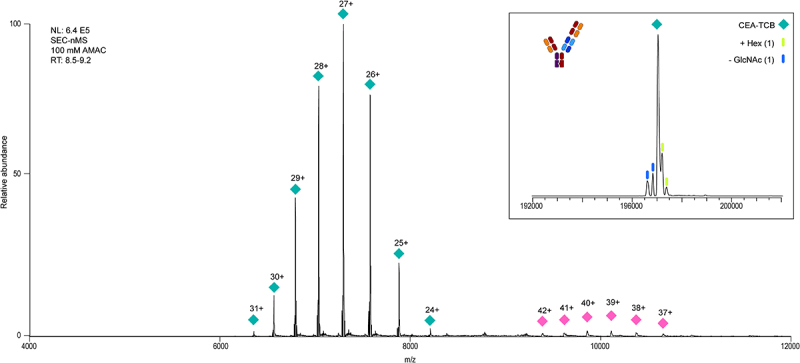
SEC-nMS Analysis of the 2 + 1 CrossMAb Product. The mass of the analyzed product is 197,037.9 Da (cyan diamond). An inset shows the charge deconvoluted spectrum from 6000 to 8500 m/z. Gains and losses of Hex(1) and GlcNAc(1) are annotated by blue and green bars. A small portion of dimer is observed, likely formed through non-covalent interactions during storage.

Next, we analyzed the 2 + 1 CrossMAb product, HMW1 and HMW2 fractions and tetravalent control by online SEC-nMS using a UHMR Orbitrap MS to further characterize the (unknown) by-products. The 2 + 1 CrossMAb (Figure 4) main product that eluted at a retention time (RT) of 8.60 to 9.00 min (Supplementary Figure 2) provided an experimental mass of 197,037.0 Da, matching closely the theoretical mass of 190,731.6 Da with 2× G0F glycans (mass difference of 6.3 Da). The charge-deconvoluted spectrum (Figure 4, inset) shows the mass shift of 1–2 hexoses attributed to the earlier reported glycation or absence of GlcNAcs, as also seen on similar antibodies.^31^ Subsequent deglycosylation by PNGase F removed the two G0F glycans from the most abundant peak, leaving only three low abundant mass shifts of +162 Da, which we annotated to be linked to lysine glycation (Supplementary Figure 3). To confirm the masses of the dimer, we analyzed the HMW2 fraction by SEC-nMS (Supplementary Figures 4–5). SEC-nMS revealed a mass of 394,084.8 Da, an acceptable mass difference of 21.9 Da compared to the theoretical mass of 394,062.9 Da. The SEC-nMS spectra obtained from this fraction (Supplementary Figure 4 and 6) also showed the presence of the main product and the products with a loss of two LCs (145 kDa) and a dimer of the two unique LCs. As described in the mass photometry results section, we could not dissociate the CrossMAb dimer during mass photometry analysis under acidic conditions, but we did observe dissociation under denaturing conditions on SDS-PAGE. This suggests that certain dissociation happened after initial separation by SEC before native MS analysis. The stable nature of the dimer and observed partial dissociation provide a clue as to why the biological activity of this fraction is lower than that of the 2 + 1 CrossMAb. The dimer can be either covalently bound, which may hinder binding, or non-covalently bound and unstable.

### SEC-native MS analysis of by-products in the deglycosylated HMW1 fraction

**Figure 5.**
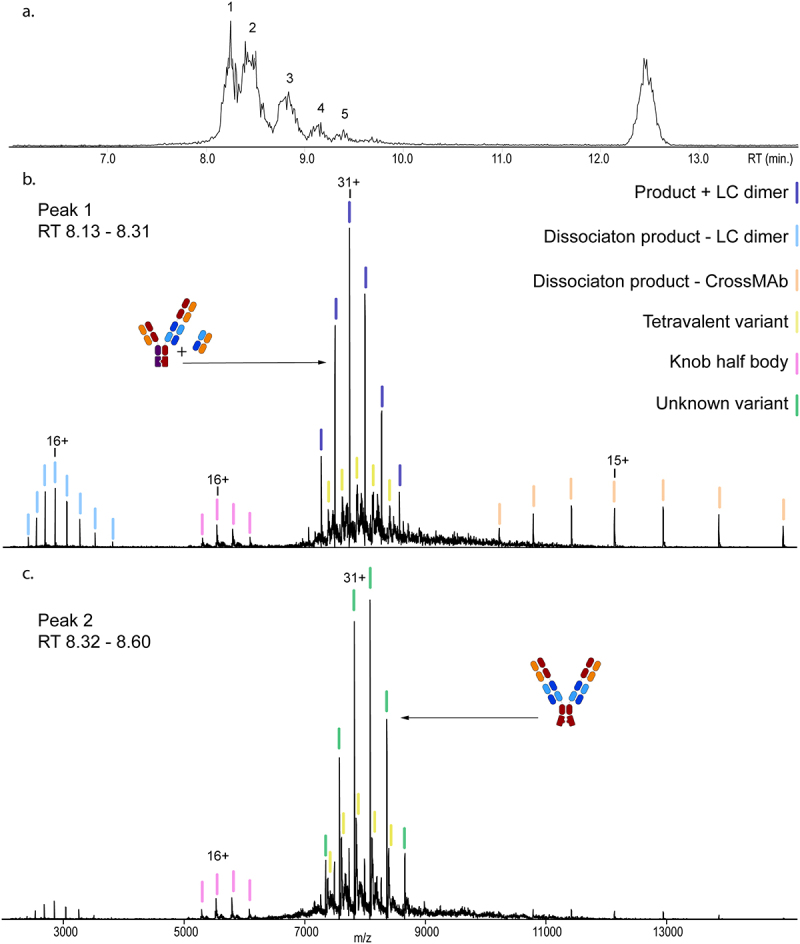
SEC-nMS Analysis of the HMW1 Fraction after Deglycosylation by PNGase F Treatment. a. The base peak chromatogram of the fraction, with six distinct peaks representing the high MW variants, the product, product fragments and the light chains. b. Native mass spectrum of peak 1, with a 2 + 1 mAb – LC-dimer product as the most abundant species (dark blue). The knob-knob variant is present in low abundance (violet), as is its half body (pink). The two series in light blue show the gas-phase disassociation products of the most LC-dimer variant broken into the LC dimer and the TCB MAb. c. Native mass spectrum of peak 2, containing an unknown size variant (green) as the most abundant proteoform and a relatively higher abundant knob-knob variant, displaying high heterogeneity. The LC-dimer and the disassociation products are nearly no longer present.

We confirmed the HMW1 fraction contained more than just the tetravalent variant because SEC displayed multiple chromatographic peaks (Figure 5a, peaks 1–5). HMW by-products were observed in peak 1 and 2. The corresponding native MS spectra revealed a highly heterogeneous mixture of by-products, even after deglycosylation (Supplementary Figure 7). In the first chromatographic peak (peak 1, RT 8.1–8.3 min.) the most abundant species is a previously unseen by-product with a mass of 239,715.6 Da (Figure 5b). This species closely matches the mass of the 2 + 1 CrossMAb product with an extra LC_xy_ dimer attached (theoretical mass of 239,707.9 Da, 7.7 Da difference). Further validating our assignment using tandem MS (i.e., higher energy C trap dissociation, HCD), this species dissociates to form an LC_xy_ dimer of 45,565 Da and the 2 + 1 CrossMAb of 194,146 Da. The facile formation of the LC_xy_ dimer in these experiments hints at its non-covalent binding to the 2 + 1 CrossMAb product. Co-eluting heavier unknown by-products were observed (Figure 5b), none matching the theoretical mass of the tetravalent variant (242,702 Da).

Tetravalent by-products were more prominent in chromatographic peak 2 (RT 8.32–8.60 min.) than in peak 1. We also observed another highly abundant, homogeneous, unknown by-product (Figure 5c). Based on its observed mass of 242,427.8 Da, we could not directly annotate this by-product using any combination of fragments or PTMs. Also, in this peak, the tetravalent variant was found with an observed mass of 242,737.6 (35.6 Da difference). Directly following the proteoform representing the tetravalent by-product is a region of high heterogeneity with multiple peaks spanning a mass range of almost one kDa. This high heterogeneity from unknown modifications rendered us unable to annotate any peak of significant meaning. However, we conclude that compared to the 2 + 1 CrossMAb product, HMW by-products substantially increase sample complexity despite being produced under the same conditions. This increased heterogeneity seemed inherent to the tetravalent variant and could relate to the non-covalent binding nature of these species. Unsuccessful binding of the Fc region would expose free cysteines and potentially lead to other modifications.

We also observed the 2 + 1 CrossMAb product and the 2 + 1 CrossMAb – LC_xy_ dimer (Supplementary Figure 8). We also detected the bivalent knob half-body with a mass of 121,340.8 Da (mass difference of 10.1 Da). The knob half-body likely originates from the unstable tetravalent by-product, owing to its apparent heterogeneity. Its presence confirms that the tetravalent variant is partially non-covalently bound and the main contributor to the high heterogeneity. The product + LC_xy_ dimer by-product and the unknown by-product also represent the majority of species in the fractions, implying that these by-products do not have an increased potency.

### Elucidating the unknown by-product

**Figure 6.**
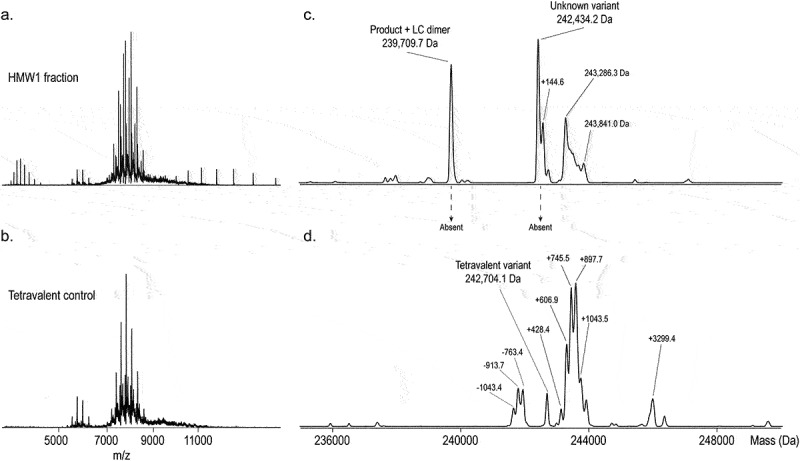
Side-by-Side Comparison of SEC-nMS of the HMW1 Fraction and the Tetravalent Control after Deglycosylation. Two by-products in the HMW1 fraction are absent in the tetravalent control. The heterogeneity annotated to the tetravalent variant-product are present in both. a. Raw native spectrum of HMW1. b. Raw native spectrum of the tetravalent control. c-d. Charge deconvoluted spectra of the HWM1 (c) and tetravalent control (d) narrowed in on the region of the high MW variants (235–250 kDa). Mass shifts are indicated but were not annotated.

The presence of the tetravalent variant was confirmed with a side-by-side comparison to the tetravalent control (Figure 6). In the SEC chromatogram of the control sample, only one other species elutes – the knob half-body (Supplementary Figure 9). In the control sample, we can get a more accurate mass annotation of the tetravalent by-product with an observed mass of 242.703.9 (2.0 Da mass difference). We observed that other by-products are not present in the tetravalent control (Figure 6c-d). As such, we confirm the heterogeneity to be inherent to the tetravalent variant. The unknown by-product in the HMW1 fraction is not present in the tetravalent control. Based on its proximity in mass to a tetravalent assembly, we expected it to be a stable, truncated tetravalent variant. To test this, we set up a CD3 affinity column online-coupled to native MS according to Lippold et al.,^32^ which separates by-products based on the amount of CD3 Fabs, thus separating trivalent from tetravalent variants as shown in Supplementary Figure 10. We then observed the unknown by-product to elute at a retention time indicating the presence of two CD3 Fabs (Supplementary Figure 10). We thus concluded this species to be a truncated tetravalent variant of high abundance in the HMW1 fraction, with a different biological activity. From this, we conclude that unwanted activity only arises when a large amount of tetravalent by-products is present, which agrees with the data from the spike-in activity assays described above.

Although we acquired more insight into the nature of these by-products, we did not yet annotate the highly heterogeneous proteoform region associated with the tetravalent variants. So far, it is known that these complex antibody formats can show unexpected Fab glycosylation, N-terminal modifications and more.^23^ Yet, these modifications cannot account for all mass shifts associated with the tetravalent by-products in the HMW1 fraction. To localize the heterogeneity, we incubated the fraction (glycosylated) with FabDELLO to remove the Fab region and assigned all heterogeneity to the Fc region (Supplementary Figure 11). As such, we propose that the random linkage of two bivalent knob-half bodies is either a cause or effect of the heterogeneity observed. In one case, non-covalent interactions could occur between different parts of either Fc region, forming unstable, randomly linked tetravalent by-products. In other cases, this would mean steric hindrance from two knob sites prevents proper disulfide linkage. Free cysteines then react with molecules present in the bioreactor and the Fc region would be exposed to other modifications.

### Bottom-up MS analysis

Next, to annotate the observed mass shifts in the HMW1 fraction, we performed both reducing and non-reducing peptide mapping on the 2 + 1 CrossMAb product, the tetravalent control and the HMW1 fraction. We noticed differences in the peptide mapping under reducing conditions between the HMW1 fraction and the 2 + 1 CrossMAb product (Suppl. Table 2) that could explain some of the heterogeneity. For example, the abundance of the signaling peptide remaining attached was higher in the HMW1 fraction. At the C-terminus, the HMW1 fraction has slightly more lysine additions and the tetravalent control a large increase, showing the tetravalent variant to be more modified. Conversely, the HMW1 fraction had an increase in C-terminal proline amidation. Also, when glycosylated, the HMW1 fraction by-products reveal aberrant glycosylation in the Fc region with N-glycan branching, sialylation and fucosylation. This adds insight to the findings of the (glycosylated) FabDELLO digests, where heterogeneity of the by-products was localized to the Fc region. We report here only the most frequent differences in modifications.

Then, based on the non-reducing peptide mapping, we annotated many cysteine modifications in the HMW1 fraction. Cysteinylation (+119 Da) and gluthationation (+305 Da) of the cysteines were increased in the HMW1 fraction and tetravalent control. Because relative quantification is difficult when comparing non-reduced peptides, absolute amounts of cysteine-modified peptides were counted. In HMW1 and the tetravalent control, these modifications were found mostly in the hinge region and knob region of the knob HCs. This finding is directly in line with the observed non-covalent binding of tetravalent variants. Although we cannot annotate all masses in the native MS spectra, we can, for example, annotate +128 and +256 Da mass shifts based on the identified lysine modifications. Finally, all annotated and observed masses for each sample were collected in Supplementary Table 3.

By combining native MS and peptide mapping data, we could better annotate some of the mass shifts from the tetravalent by-products. In the HMW1 fraction, the unknown tetravalent by-product is the most abundant species, reducing the resolution of the proteoform peaks of the other tetravalent by-products. However, compared to the tetravalent control, most masses remained unannotated due to the low resolution. To simplify the annotation, we compared the half-bodies of the HMW1 by-products and control (Supplementary Figure 12). The knob half bodies from the HMW1 fraction and the tetravalent control revealed similar masses and mass shifts. Although we were unable to annotate all mass shifts in the tetravalent by-products, we identified the source and localization of heterogeneity in HMW by-products and the PTMs contributing to this heterogeneity.

From our data, we extract two important findings. First, the production of the 2 + 1 CrossMAb product leads to three distinct by-products. One of these by-products, the tetravalent variant, exhibits a high degree of heterogeneity primarily responsible for the analytical complexity of the HMW1 fraction. Second, a significant portion of the HMW by-products is non-covalently bound, allowing for heavily modified assemblies and decreasing biological activity. This explains the discrepancy in activity between the HMW1 fraction and the tetravalent control. Using SEC-nMS, these by-products could be rapidly identified based on their mass and the risk assessed based on our observations in the activity assays. Additionally, our results can help analytical method development for other complex antibody formats present HMW by-products. Based on our data, we can also predict new types of by-products during a product development process.

## Discussion

A multi-method approach was used to characterize a complex trivalent bsAb and its HMW by-products. The characterization was linked to the biological activity of the product and by-products. Most useful was the use of native MS for rapid identification of species present in a SEC fraction. This characterization was complemented by mass photometry and SDS-PAGE to investigate non-covalent binding and peptide mapping to identify PTMs associated with the by-products of concern. Initially, we expected that the dimer aggregate of the 2 + 1 CrossMAb would portray increased potency. However, it was much less active than the product. When formed, the dimer aggregate might prevent effective interaction with the antigen. Partial covalent binding and different assemblies are also seen in standard mAbs and can be related to their activity.^33^ Instead, we found that the tetravalent variant showed an increase in potency in activity assays, as it was more potent than the 2 + 1 CrossMAb product. Even more surprising, the HMW1 fraction collected with SEC, expected to contain this tetravalent variant, showed similar potency to the product.

Although fully purified 2 + 1 CrossMAb product is already highly pure (Figure 1), it is always crucial to have a good understanding of low abundant by-products. With the approach presented here, we provide a range of strategies to answer questions about the chemical binding nature or structures and modifications of HMW by-products. In addition, we use well-described methods.^13,29,34,35^ Our multi-method approach helped explain differences in potency and characterize by-products formed during production. We were able to show by SDS-PAGE that there are multiple and non-covalent by-products in the HMW1 fraction. These results were complemented by mass photometry. By simply adding acid and comparing photometric data to the SDS-PAGE, we saw the partial non-covalent nature of the tetravalent variant. Mass photometry is a handy and rapid tool to give insight into therapeutics and by-products under different conditions and is already finding its place in the analysis of therapeutics.^29^ It allows the manipulation of the environment by introducing other buffers and reducing agents and ligands all within minutes per measurement. Another advantage is that this approach is not limited to only complex mAb formats. This also includes effects such as polymerization and aggregation, of which a recent example is the investigation of the multiple forms of haptoglobin.^22^ The main disadvantage of mass photometry is the lower resolution when analyte mass differences are below ~20 kDa.

Similarly, native MS is generally useful for the characterization of antibodies, but was in our case greatly complemented by separation before measurement. SEC-nMS compared to direct injection successfully separated the highly heterogeneous HMW1 fraction enough to directly annotate three major distinct by-products. We could annotate two based on their mass, namely the product + LCx_y_ dimer and the tetravalent variant. The third could not be annotated by its mass alone, as it was about 280 Da below the mass of the tetravalent variant. However, a different separation using CD3 affinity enabled us to determine that this species was in fact also a tetravalent variant. Native MS has already found its place in the analysis of mAbs. Previously it was successfully applied to non-covalent antibody/receptor complexes and other bsAb size variants.^8^ Recent developments in native MS using cation exchange chromatography (CEC)-MS now also allow for the characterization of multiple PTMs by the separation of charge variants,^23^ applicable for identifying or monitoring asparagine deamidation, primary and secondary Fab glycosylation and O-glycosylation. Online-coupled SEC-nMS is also gaining in popularity.^34^ The advantage of native MS over routine testing methods such as SEC-HPLC, ion-exchange (IE)-HPLC and capillary electrophoresis (CE) is the increased resolution and the significant increase of in-depth information gained on a biotherapeutic.

One question that remains partially unsolved is the annotation of all proteoform peaks seen for the tetravalent by-products. Although we were able to locate the heterogeneity to the Fc region by enzymatic cleavage of the Fab region, direct annotation was impossible due to the amount of heterogeneity. However, the combination of reducing and non-reducing peptide mapping allowed us to identify the PTMs responsible for the heterogeneity, as well as the occupation of cysteines not involved in disulfide bonds. This is a perfect example of the increasingly complex by-products of complex antibody formats. It was already known that disulfide bonds in the hinge region and near the light chains could be missing and presented as free cysteines.^12,36^ These cysteines are then occupied by PTMs such as cysteinylation or glutathionation, or even additional light chains. In the end, we did not fully annotate all mass shifts, but improved our understanding of the by-products. For this reason, we also focused mostly on the HWM1 fraction. Most importantly, we sourced its heterogeneity to the knob HC, specifically the Fc region. Thus, we infer that the incorrect assembly of mAbs with a knob can lead to HMW variants that are subject to a range of modifications.

In this specific case, the formation of the tetravalent variant with cysteine modifications and other PTMs also hints at a potential lead for developments in protein engineering to ensure even more correct assembly, reducing the chance of such by-products forming. We identified that only the tetravalent CrossMAb was of higher potency within the HMW variants; however, spiking of up to 10% of the tetravalent variant led to no change in the potency assay. Taking into account the expectation that only the tetravalent portion of the HMW1 fraction is of increased potency, no impact of HMW variants on potency is expected. This provides an additional layer of confidence in the safety and efficacy of complex antibody therapeutics. We, therefore, suggest researchers involved in method development for complex antibody formats should consider adopting elements of our approach. Altogether, the reported findings show that a multi-method approach for the characterization of a complex 2 + 1 bsAb elucidates not only the masses and characterization of HMW by-products, but also their chemical binding nature. Based on the activities associated with by-products, we were able to assess which variants pose higher risks and how these variants differ in structure or chemical bonding. We believe that, as of now, the described methods can be directly implemented to analyze a wider range of therapeutics with HMW by-products and those with non-conventional structures, aggregates, or complex mixtures of by-products.

## Materials and methods

### Materials and chemicals

CrossMAb samples were produced in-house in CHO cell lines at Roche Diagnostics GmbH. Ammonium acetate 7.5 M solution, tris(2-carboxyethyl)phosphine, CAA, sodium deoxycholate, trypsin, PBS and glacial acetic acid were purchased from Sigma Aldrich. Materials for mass photometry were also purchased from Sigma Aldrich. Pre-cast gels (12%) were acquired from Bio-Rad.

### Potency assay

Microtiter plates were coated with the unspecified antigen and incubated for 1 hour at room temperature. After incubation, coating solution was discarded. 100 µl/well of the diluted reference standard, product control and samples were added and incubated for 1 hour at room temperature. After washing the plates, 150 µl of cell suspension were added to each well and incubated for 4 h at 37°C. After washing the plates, 50 µl of ONE-Glo™ (Luciferase Assay system, Promega) was added to each well and plates incubated for 30 min at R room temperature. The relative potency of a sample or product control was calculated using the 4-parameter parallel-curve model.

### Purification of HMW1 (By-product Mixture) and HMW2 (Dimer) by size exclusion

CrossMAb fermentation broth was purified using a MabSelect SuRe column (5 cm × 25 cm, GE Healthcare). The column was equilibrated with 25 mM Tris/HCl, 25 mM NaCl pH 7.0. Then, ~5 L of fermentation broth was loaded onto the column. Next, the column was washed with ~6 L of equilibration buffer. Elution of material was performed with ~800 ml of 150 mM Acetic Acid. The eluate was than pH adjusted to pH 5.0 using 1 M Tris pH 11.0 following buffer exchange to 20 mM His/His/HCl, 140 mM NaCl, and pH 6.0). The sample was then further purified by a size exclusion column (Superdex 200 height 690 mm; diameter 100 mm) to collect for HMW1 (by-products) and HMW2 (Dimer) variants. The collected variants were concentrated using centrifugal devices (Amicon Ultra – 15; Centrifugal Filters; Ultracel – 30 K) and buffer exchanged to 20 mM His/HCl, 140 mM NaCl, pH 6.0.

### Fermentation and purification of tetravalent CrossMAb variant by size exclusion

For reference purposes, a tetravalent CrossMAb reference was produced in human embryonic kidney (HEK) cells. 5 L of fermentation broth was purified using a MabSelect SuRe column (1 cm x 9.5 cm, GE Healthcare). The column was equilibrated with 25 mM Tris/HCl, 25 mM NaCl pH 7.0. Then ~5 L of fermentation broth was loaded onto the column. Next, the column was washed with ~6 L of equilibration buffer. Elution of tetravalent CrossMAb variant was performed with ~800 ml of 150 mM acetic acid. The eluate was then pH adjusted to pH 5.0 using 1 M Tris pH 11.0 following buffer exchange to 20 mM His/His/HCl, 140 mM NaCl, pH 6.0). The tetravalent CrossMAb was further purified by a size exclusion column (SuperdexTM 200 height 690 mm; diameter 100 mm) to remove unwanted by-products and purify the tetravalent CrossMAb variant. The respective CrossMAb variant peak on the size exclusion column was collected. The individual fraction was concentrated using Amicon Ultra – 15; Centrifugal Filters; Ultracel – 30 K) and buffer exchanged in 20 mM His/HCl, 140 mM NaCl, pH 6.0.

### Non-reducing SDS-PAGE

The following aliquots of samples; 5 µg of trastuzumab (Herceptin); 10 µg of the 2 + 1 CrossMAb, HMW1 fraction, HMW2 fraction and tetravalent control were added up to 20 µL of Bio-Rad 1x sample buffer and heated for 10 minutes at 60 C°. The procedure was performed according to the vendor’s instructions, with a runtime of 30 min at 30 mA, and 4.5 hours at 50 mA.

### Mass photometry analysis

Borosilicate coverslips and silicon gaskets were washed and cleaned with isopropanol and Milli-Q water. Silicon gaskets were stuck on the coverslips to hold samples during analysis. A Refeyn OneMP instrument was used for the analysis and calibrated using a native marker protein standard mixture (NativeMark Unstained Protein Standard, Thermo Scientific), containing proteins in a mass range from 20 to 1200 kDa. A calibration curve was generated using the following masses: 66, 146, 480 and 1048 kDa. The 2 + 1 CrossMAb product, HMW1 and HMW2 fractions and the tetravalent control were prepared by diluting 200–2000 times, adding 2–5 µL of sample into 9–13 µL of buffer in the gasket to a final concentration of 4–30 nM. The buffer was PBS or 0.2 M glacial acetic acid. Using AcquireMP software, movies of particle landing events were acquired per sample for 9000 frames at 100 frames per second. Between 500 and 5000 particle-landing events were detected per sample. Data was processed in DiscoverMP software, estimating masses of the samples by the mode of a Gaussian distribution fitted on mass histograms. Kernel density plots were exported as data.

### Native MS (SEC directly coupled to native ESI-MS)

SEC directly coupled to native ESI-MS (native SEC-UV/MS) was carried out using an ACQUITY UPLC Protein BEH SEC column (4.6 × 300 mm, 1.7 μm particle size; Waters). An isocratic elution using 100 mM CH_3_COONH_4_, pH 7.0 at 0.2 mL/min was used for chromatographic separation on a Vanquish Horizon UHLPC system (Thermo Fisher Scientific) equipped with UV detection at 280 nm. Samples were adjusted to a concentration of 2 μg/μL in 0.15 M sodium phosphate pH 7.0 by diluting 100 μg of CrossMAb in a total volume of 50 μL. Sample injection amounts of 20 μg CrossMAb were used and data acquisition was controlled by Chromeleon software (Thermo Fisher Scientific). The outlet of the Vanquish Horizon UHLPC system was directly coupled to the Nanospray Flex ion source (Thermo Fisher Scientific). The RSLC flow was split postcolumn with 7 μL/min directed to the MS system and 293 μL/min disposed to waste. The Nanospray Flex ion source was installed on a Thermo Scientific UHMR (enhanced mass range) mass spectrometer (Thermo Fisher Scientific). The UHMR mass spectrometer was operated in positive ion mode (m/z 2000 − 15,000) and the resolution of the Orbitrap mass analyzer was set to 12,500. The capillary voltage was set to 2.6 kV and in source collision energy to 30.0 eV.

### CD3 Target affinity chromatography mass spectrometry

CD3 target affinity chromatography with MS was performed according to Lippold et al.^32^ Initial tests on CD3 affinity columns were conducted using MS non-compatible mobile phases and UV-only detection (absorbance at 280 nm) (Information S2). MS-compatible mobile phases consisted of 200 mM ammonium formate (mobile phase A) and 200 mM formic acid (mobile phase B). All experiments using MS detection were performed on a Vanquish Horizon (Thermo Scientific). The column temperature was set to 35°C and the flow rate was kept at 0.25 mL/min. Prior to MS experiments, samples were buffer-exchanged to mobile phase A (final conc. 1 mg/mL) using 10 kDa spin-filters (Merck). The injection volume was set to 10 µL (10 µg). Prior to the electrospray ionization (ESI), flow-splitting was applied, directing around 2 µL/min to the nanospray Flex ion source (Thermo Scientific). A stepped pH gradient was used for elution. The first 5 min were kept at 100%A, following a linear gradient to 50% at 9 min. An additional linear increase to 90% B at 20 min was applied. In addition, a washing step (90% B) was performed for 5 min. Re-equilibration of the column was achieved using 100% A for 15 min. For MS detection, a Q Exactive UHMR Orbitrap (Thermo Scientific) was used. MS data was acquired in an m/z range of 2,000 Th to 15,000 Th. The resolution of the instrument was set to 12,500. Capillary voltage was set to 2 kV (positive ion mode), in-source collision-induced dissociation energy to −30 V, and HCD energy to 175 V. 10 microscans were averaged for each data point. Proteinmetrics software (PMI) was used for analyzing intact mass data. Charge states from 5 to 35 and a mass range of 25 kDa to 250 kDa were applied for deconvolution in relevant retention time windows. The signal intensity of deconvoluted MS spectra was used to estimate relative abundances of proteoforms.

### Native MS Data analysis

Theoretical masses were calculated based on the amino acid sequence with corrections for known PTMs such as c-terminal lysine cleavage, Glu to pyroGlu conversions, glycosylation and disulfide bonds. Masses of individual proteoforms of the fractions were acquired by zero-charge state deconvolution of the native MS-spectra using PMi Intact Mass software (ProteinMetrics, version 3.3). All resulting data was manually checked with the corresponding raw spectra.

### Data evaluation of variants at the intact protein level for SEC-UV/MS and affinity CD3 column

For the intact and FabALACTICA (IgdE) digest, intact mass data analysis was performed using the PMi Intact Mass software for mass determination of respective peaks in the total ion chromatogram (TIC). The m/z mass signals were deconvoluted and annotation of peaks was carried out based on the protein sequence in combination with a manually created delta mass list of possible modifications. The relative quantification of protein variants was performed using the absolute intensity of the deconvoluted mass signals.

### Non-reduced enzymatic peptide mapping with LC-MS/MS

For all samples, 5 µg of protein was digested using trypsin or Glu-C at a 1:75 enzyme:protein ratio (w/w) overnight at 37°C in duplo. Following digestion, samples were desalted using Oasis microElution 96–well plates (Waters, Wexford, Ireland) as previously described,^37^ dried and dissolved in 0.1% formic acid (FA). About 100 ng of peptides was separated and analyzed using an Agilent 1290 Infinity HPLC system (Agilent Technologies) coupled online to an Orbitrap Fusion mass spectrometer (Thermo Fisher Scientific) as previously described^38^ using HCD and electron-transfer/higher-energy collision dissociation (EThcD) as fragmentation methods. Data was analyzed using Byonic software. Raw data was searched against the known sequence, specifying the corresponding protease. Modifications included in the search were oxidation, pyro-glutamate formation, lysine glycation, glutathionation, cysteinylation and disulfide linkage to other peptides. The abundance of cysteine modifications was reported by comparing between samples the number of peptides reported with a cutoff score of 100. All counted spectra were checked manually.

### Reduced enzymatic peptide mapping with LC-MS/MS

Tryptic Peptide Mapping for the detection and quantification of Asn deamidation, Fab glycosylation, O-xylose variants, Lys glycation, and N- and C-terminal modifications at peptide level, CrossMAb reference material, and preparative SEC fractions were denatured in 0.4 M Tris/HCl, 8 M Gua, pH 8.5 by diluting 350 μg of CrossMAb in a total volume of 300 μL. For reduction, 10 μL of 0.1 g/mL dithiothreitol (DTT) was added, followed by incubation at 50°C for 1 h. After alkylation of free cysteines by adding 0.33 g/mL iodoacetic acid and incubation at room temperature in the dark for 30 min, the buffer was exchanged to digestion buffer (20 mM His/HCl, pH 6.0) by application onto a NAP-5gel filtration column. Subsequently, the NAP-5 eluate (500 μL) was mixed with 10 μL of a 0.25 mg/mL trypsin solution (Trypsin Proteomics grade, Roche, #03708985001) in 10 mM HCl and incubated at 37°C for 17 h.16 The digest was stopped by adding 50 μL of a 10% trifluoroacetic acid solution. Prior to LC-MS analysis, digests were diluted 1:1 with ultrapure water and ~4 μg (20 μL) injected onto the reversed-phase (RP) column for LC-MS analysis.

### Analysis of proteolytic tryptic peptides

The tryptic peptide mixture was separated on a RP C18 column (BEH C18 1.7 μm, 2.1 × 150 mm; Waters) using a UltiMate 3000 RapidSeparation LC (Thermo Fisher Scientific) and analyzed online with an Orbitrap Fusion Tribrid electrospray mass spectrometer (Thermo Fisher Scientific). The mobile phases consisted of 0.1% formic acid in water (solvent A) and 0.1% formic acid in acetonitrile (solvent B). The chromatography was carried out using a gradient from 1 to 35% solvent B in 45 min and finally from 35 to 80% solvent B in 3 min using a flow rate of 300 μL/min. UV absorption was measured at a wavelength of 220 nm. A sample amount of ~4 μg digested protein was applied. The RSLC (Rapid Separation Liquid Chromatography) system and mass spectrometer were connected by PEEK capillary tubing. Data acquisition was controlled by the Orbitrap Tribrid MS Series Instrument Control Software Version 3.4 (Thermo Fisher Scientific). Parameters for MS detection were adjusted according to existing knowledge gained from experience with peptide analysis of recombinant antibodies.

### Data analysis reduced enzymatic peptide mapping with LC-MS/MS

Peptides of interest were identified by searching manually for their m/z values within the mass spectrum and quantified by PMi Byologic (Protein Metrics) software tool. For the quantification, extracted ion chromatograms (XIC) of peptides of interest were generated on the basis of their monoisotopic masses and detected charge states. The relative amounts of CrossMAb modifications were calculated from the manual integration results of the modified and unmodified peptide peaks.

## Supplementary Material

Supplemental Material

## Alphabetical list of abbreviations

*AUC* Area under the curve

*bsAbs* Bispecific Antibodies

*CEC* Cationic exchange chromatography

*CEC* Capillary electrophoresis

*CHO* Chinese hamster ovary

*DTT* Dithiotrheitol

*EtHCD* Electron-transfer/higher-energy collision dissociation

*FA* Formic acid

*HC* Heavy chain

*HCD* Higher energy C trap dissociation

*HEK* Human embryonic kidney

*HMW* High Molecular Weight

*IE-HPLC* Ion-exchange high powered liquid chromatography

*LC* Light chain

*mAbs* Monoclonal antibodies

*MALS* Multi-angle light scattering

*MS* Mass spectrometry

*nMS N*ative mass spectrometry

*PTM* Post-translational modification

*RP* Reversed-phase

*SEC* Size exclusion chromatography

*UV* Ultraviolet
