# Supplementary material for: Characterization of high-molecular weight by-products in the production of a trivalent bispecific 2+1 heterodimeric antibody
Source: MAbs. 2023 Feb 17;15(1):2175312. doi: 10.1080/19420862.2023.2175312 (PMC9980510; doi:10.1080/19420862.2023.2175312)
Supplement: Supplemental Material [file KMAB_A_2175312_SM8178.zip › MAbs Characterization of high molecular weight by products SI Revision 229613511.docx]

**Characterization of High-Molecular Weight By-products in the Production of a Trivalent Bispecific 2+1 Heterodimeric Antibody**

Dario A. T. Cramer^a,b^, Vojtech Franc^a,b^, Anna-Katharina Heidenreich^c^, Michaela Hook^c^, Mahdi Adibzadeh^d^, Dietmar Reusch^c^, Albert J. R. Heck^a,b^, Markus Haberger^c,*^

*^a^Biomolecular Mass Spectrometry and Proteomics, Bijvoet Center for Biomolecular Research and Utrecht Institute for Pharmaceutical Sciences, University of Utrecht, Utrecht, The Netherlands; ^b^Netherlands Proteomics Center, Utrecht, The Netherlands; ^c^Pharma Technical Development, Roche Diagnostics GmbH, Penzberg, Germany; ^d^Pharma Technical Development, F. Hoffmann-La Roche AG, Basel, Switzerland*

^*^Corresponding author: markus.haberger@roche.com

**Supporting information**

[**Supplementary table and supplements legend** 2](#_Toc106967165)

[**Supplementary figure 1** 3](#_Toc106967166)

[**Supplementary figure 2** 3](#_Toc106967167)

[**Supplementary figure 3** 4](#_Toc106967168)

[**Supplementary figure 4** 5](#_Toc106967169)

[**Supplementary figure 5** 6](#_Toc106967170)

[**Supplementary figure 6** 7](#_Toc106967171)

[**Supplementary figure 7** 8](#_Toc106967172)

[**Supplementary figure 8** 9](#_Toc106967173)

[**Supplementary figure 9** 10](#_Toc106967174)

[**Supplementary figure 10** 11](#_Toc106967175)

[**Supplementary figure 11** 12](#_Toc106967176)

[**Supplementary figure 12** 13](#_Toc106967177)

# Supplementary table and supplements legend

**Suppl. table 1:** potency assay of the 2+1 CrossMAb with different percentages spiked-in knob-knob variant

**Suppl. table 2**: relative abundances of modifications identified in reducing peptide mapping and peptides counted with cysteine modifications in non-reduced peptide mapping

**Suppl. table 3**: all observed masses of proteoforms from native MS analysis





Supplementary figure 1: **the 2+1 Heterodimeric Bispecific Antibody and its Expected By-Products.** Left: the designed 2+1 CrossMAb consists of two Fab regions against one unspecified target and one Fab region against CD3. The knob-in-hole assembly technique is highlighted in the lower part of the constant region of the heavy chain, formed by three amino-acid sequences forming a disulfide bond between the cysteines on either side. The expected HMW by-products are shown with their expected size. The tetravalent variant (middle) shows a knob-knob conformationincluding size variants (e.g. dimers), and knob-knob species with theoretical masses of around 250 to 400 kDa.


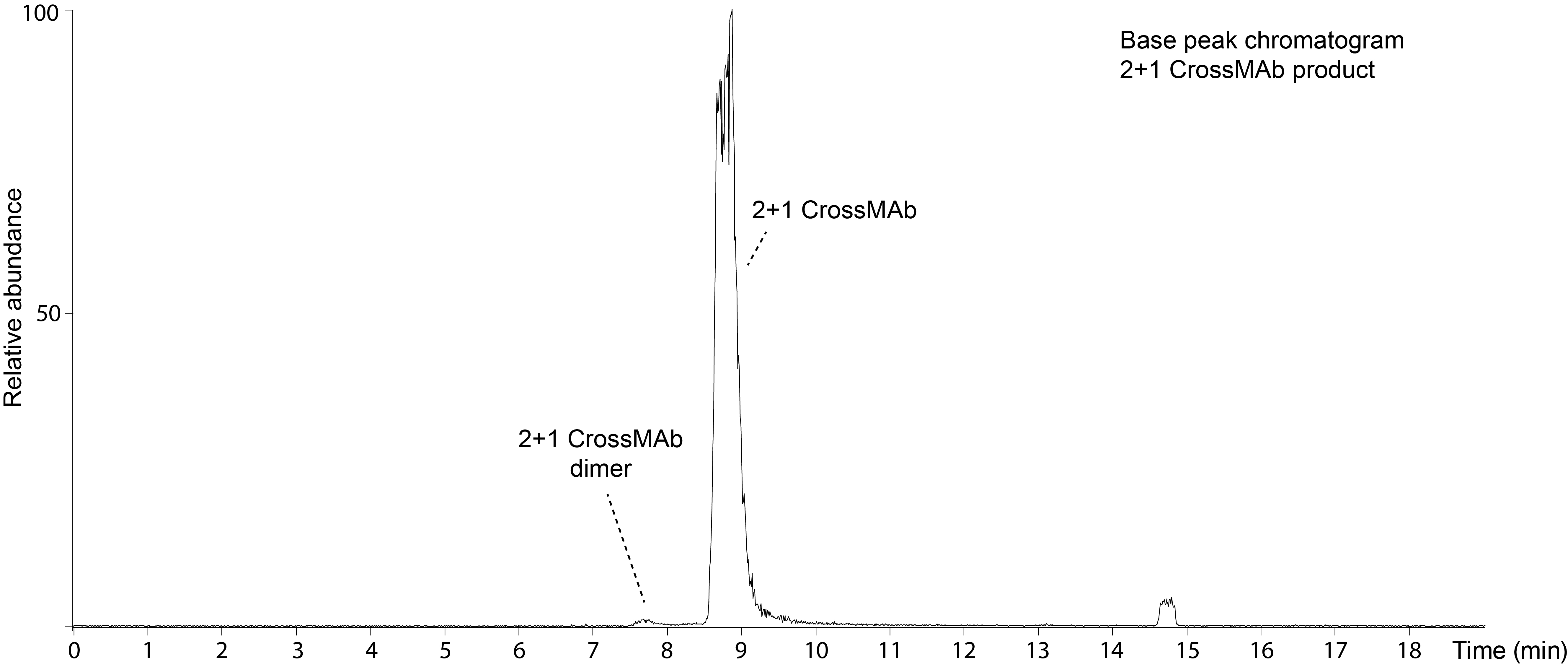


Supplementary Figure 2: **Base Peak Chromatogram of the 2+1 Crossmab.** During online coupled SEC-nMS product control elutes from 8.6 to 9.0 minutes. Eluting before that from 7.5 to 7.9 min is the dimer of the 2+1 CrossMab at low abundance.


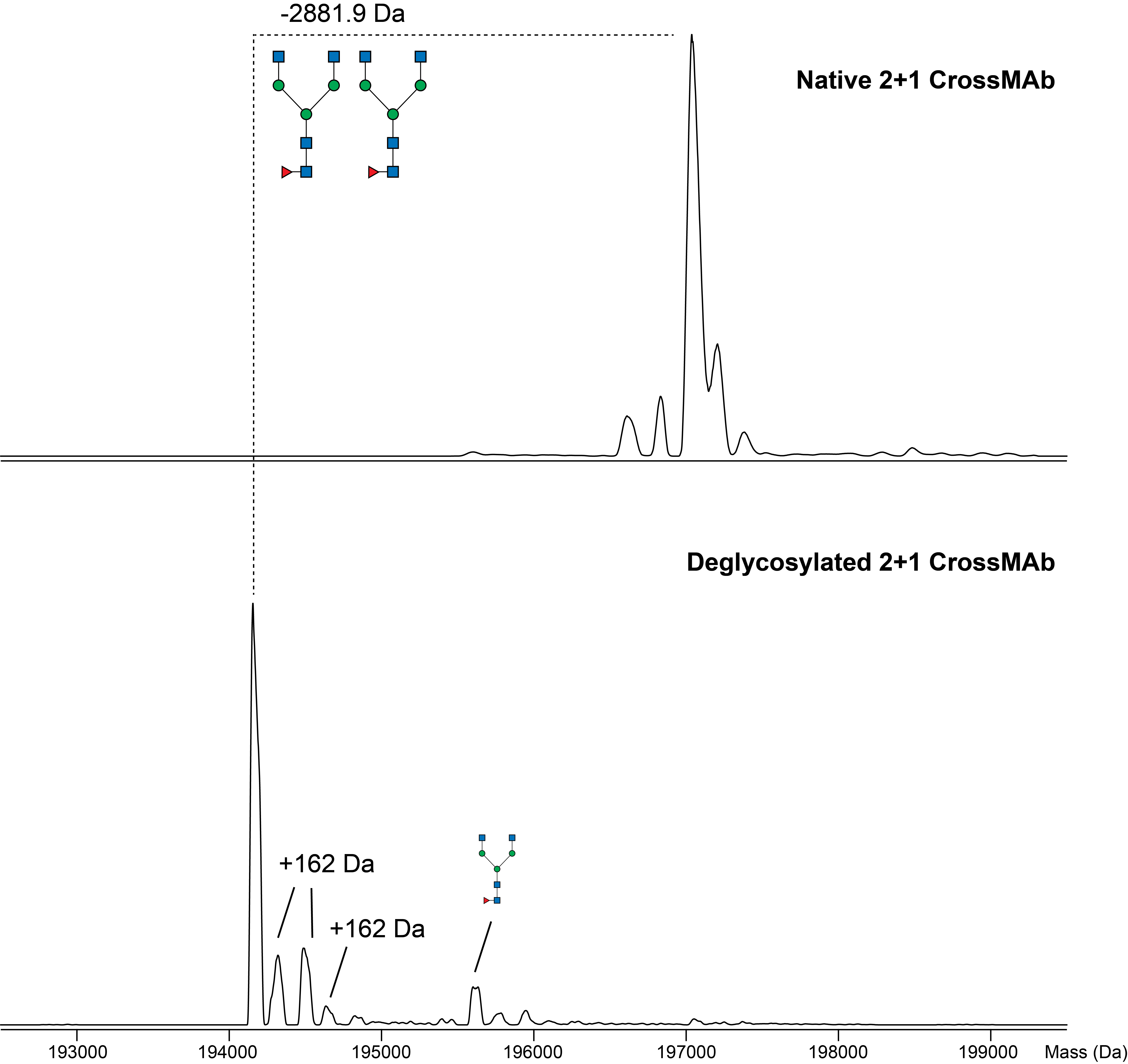


Supplementary Figure 3: **Deglycosylation of the 2+1 Crossmab.** Deglycosylation shows a loss of two G0F glycans (8.8 Da mass difference) after which a series of glycation (lysine glycation) is observed up to three times. The product is also partially deglycosylated, as shown by a low abundance proteoform corresponding to the presence of one G0F glycan.


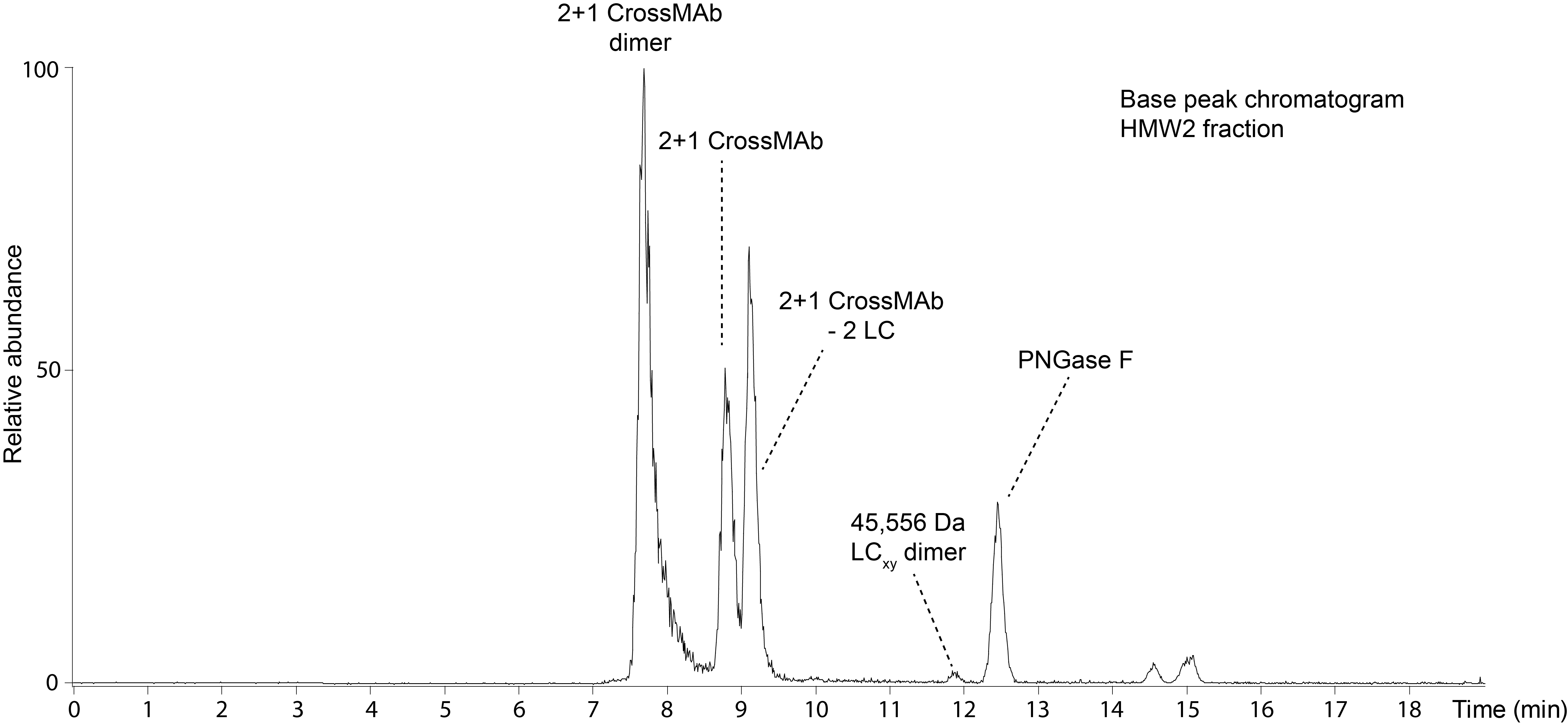


Supplementary Figure 4: **Base Peak Chromatogram of the HMW2 Fraction.** The chromatogram shows the elution of the 2+1 CrossMAb dimer during SEC-nMS analysis from 7.5-8.2 minutes. Eluting before that from 7.5 to 7.9 min is the dimer of the 2+1 CrossMab at low abundance. As indicated, the 2+1 CrossMAb missing two light chains elutes directly thereafter from 9.0-9.3 min. The final peak contains the enzyme PNGase F.


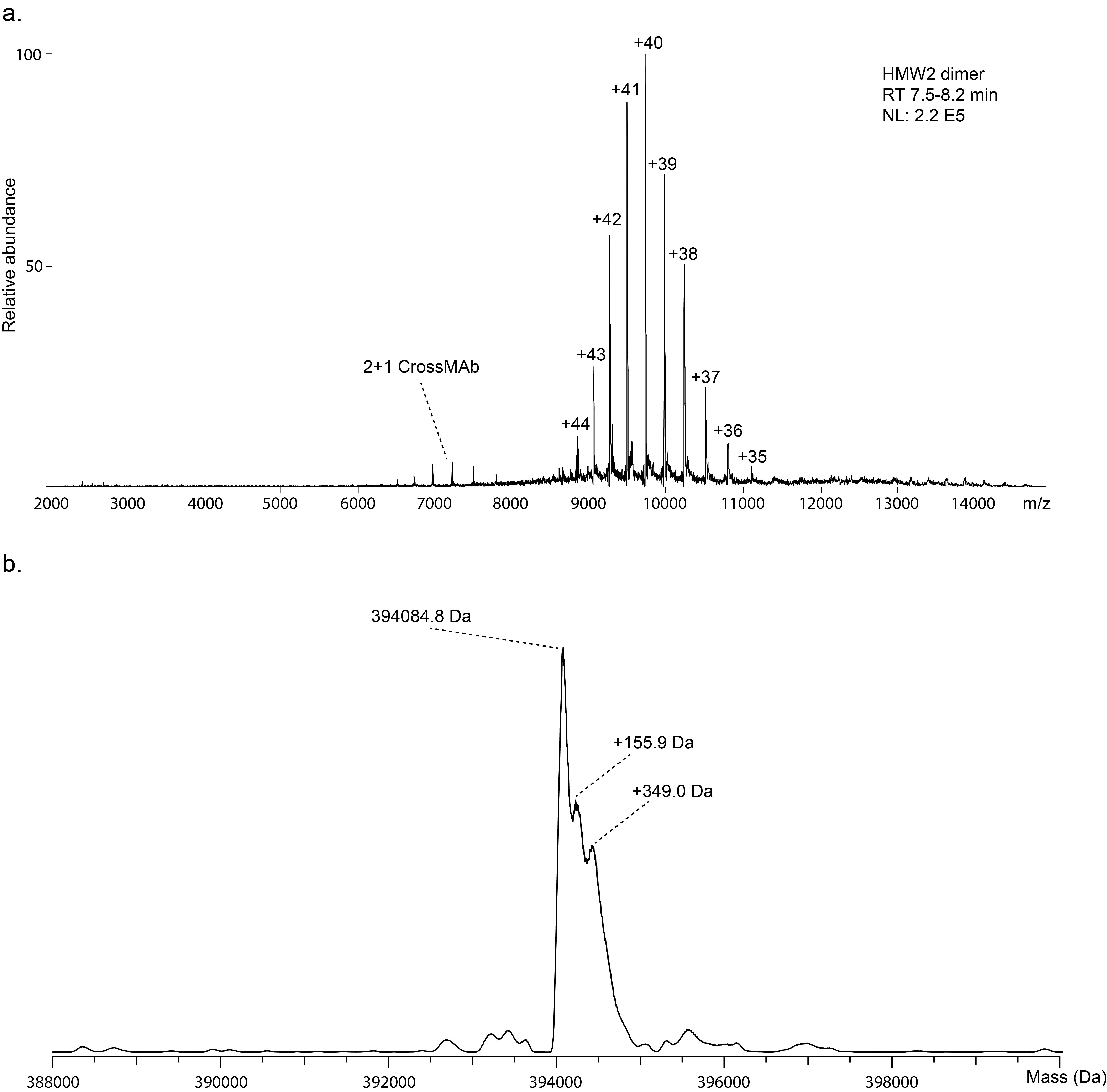


Supplementary Figure 5: **Raw and Deconvoluted Spectrum of SEC-Nms Analysis of The Deglycosylated HMW2 Fraction.** a. The dimer in the HMW2 fraction reveals a charge state envelope form +35 to +44. b. The dimer is a homogenous species corresponding to the theoretical mass, with two proteoforms not well enough resolved for annotation.


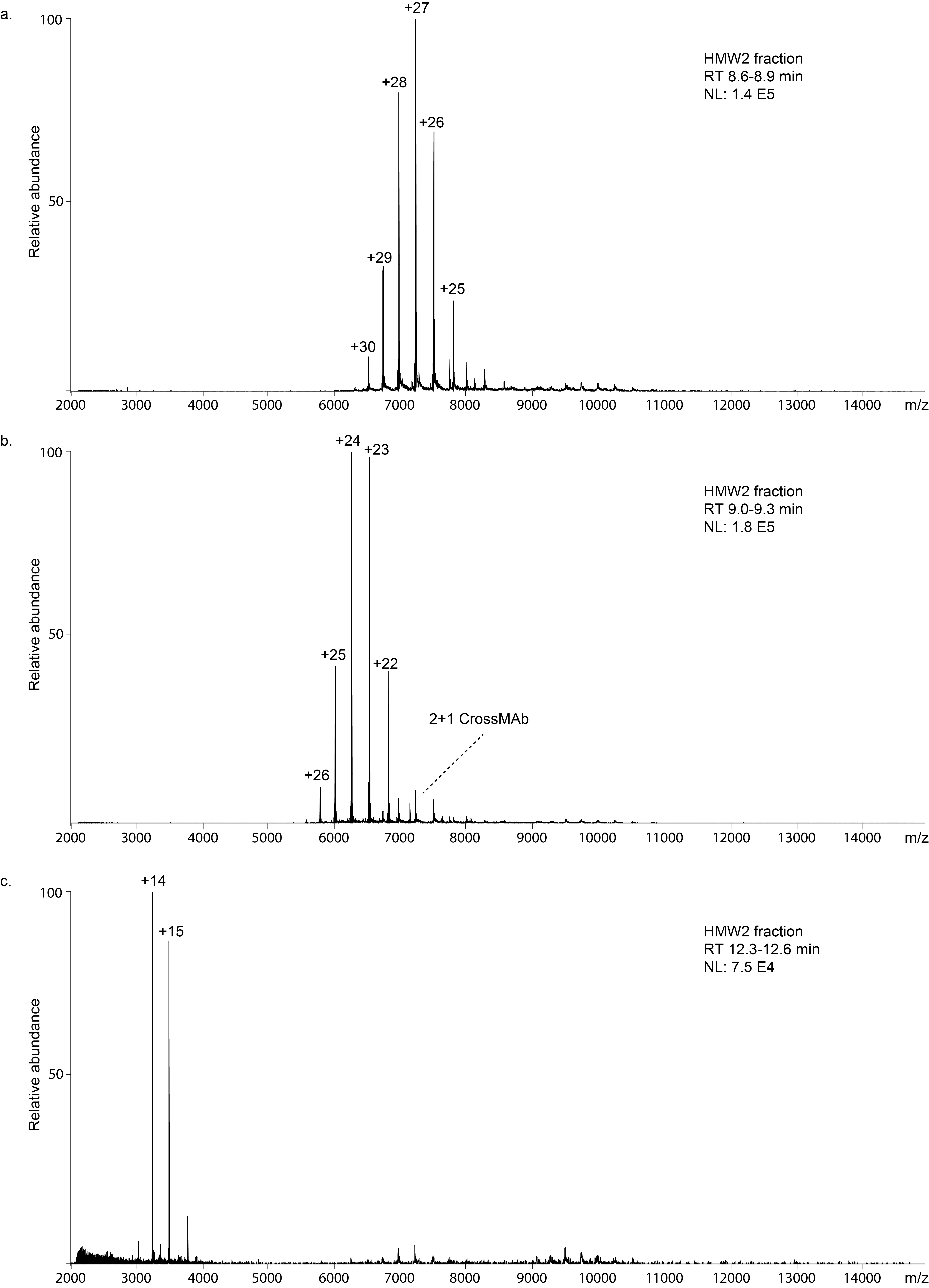


Supplementary Figure 6: **Raw Spectra of Additional Species in the Deglycosylated HMW2 Fraction.** From top to bottom, the 2+1 CrossMAb product (a), the loss of the LC_xy_ dimer with a mass of 151,463.3 Da (b) and the LC_xy_ dimer with a mass of 45,563.6 Da (c).


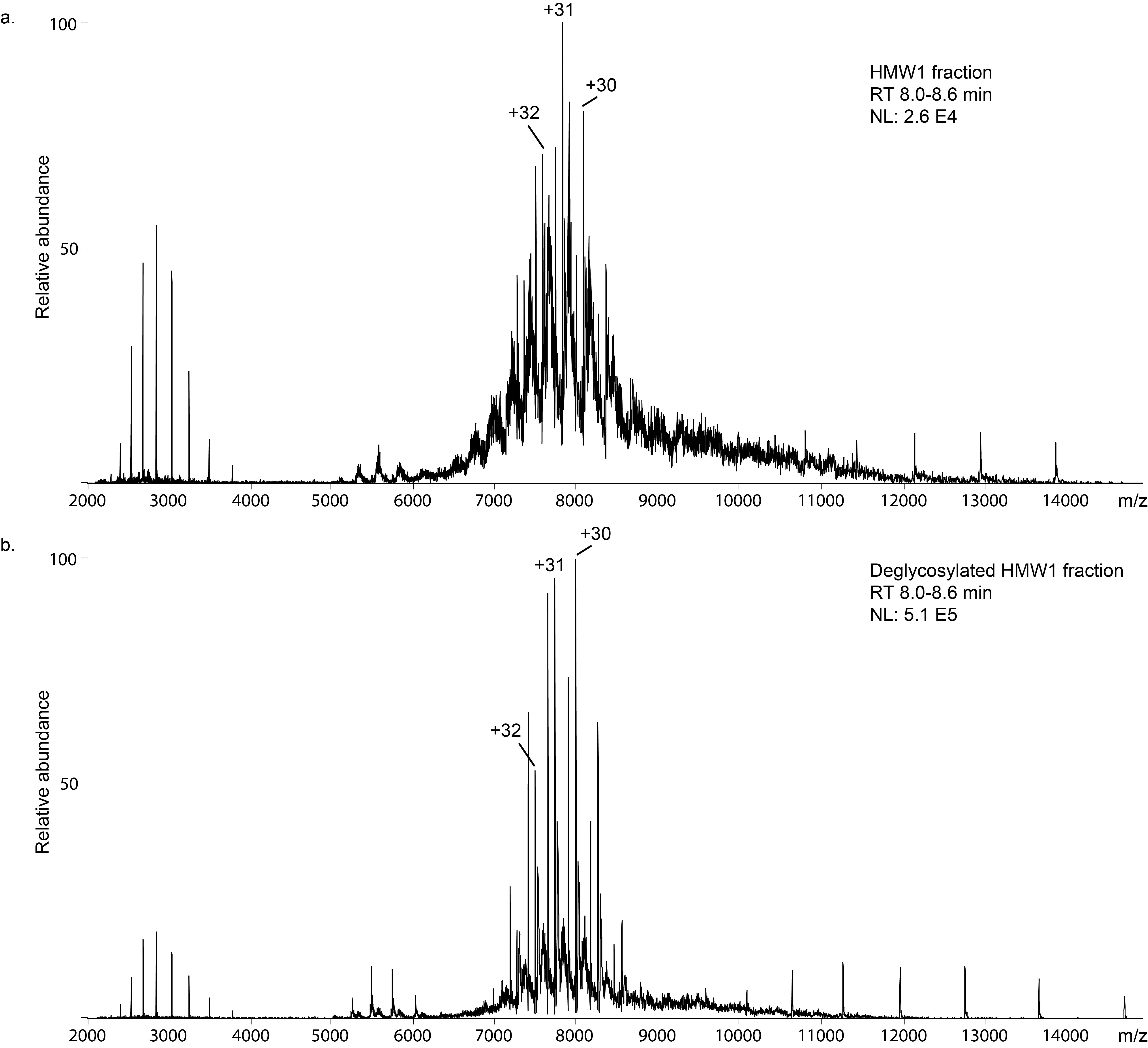


Supplementary Figure 7: **Deglycosylation of the HMW1 Fraction.** The comparison of native and deglycosylated HMW1 fraction shows that though spectral complexity is reduced, the sample stays complex and heterogeneous.


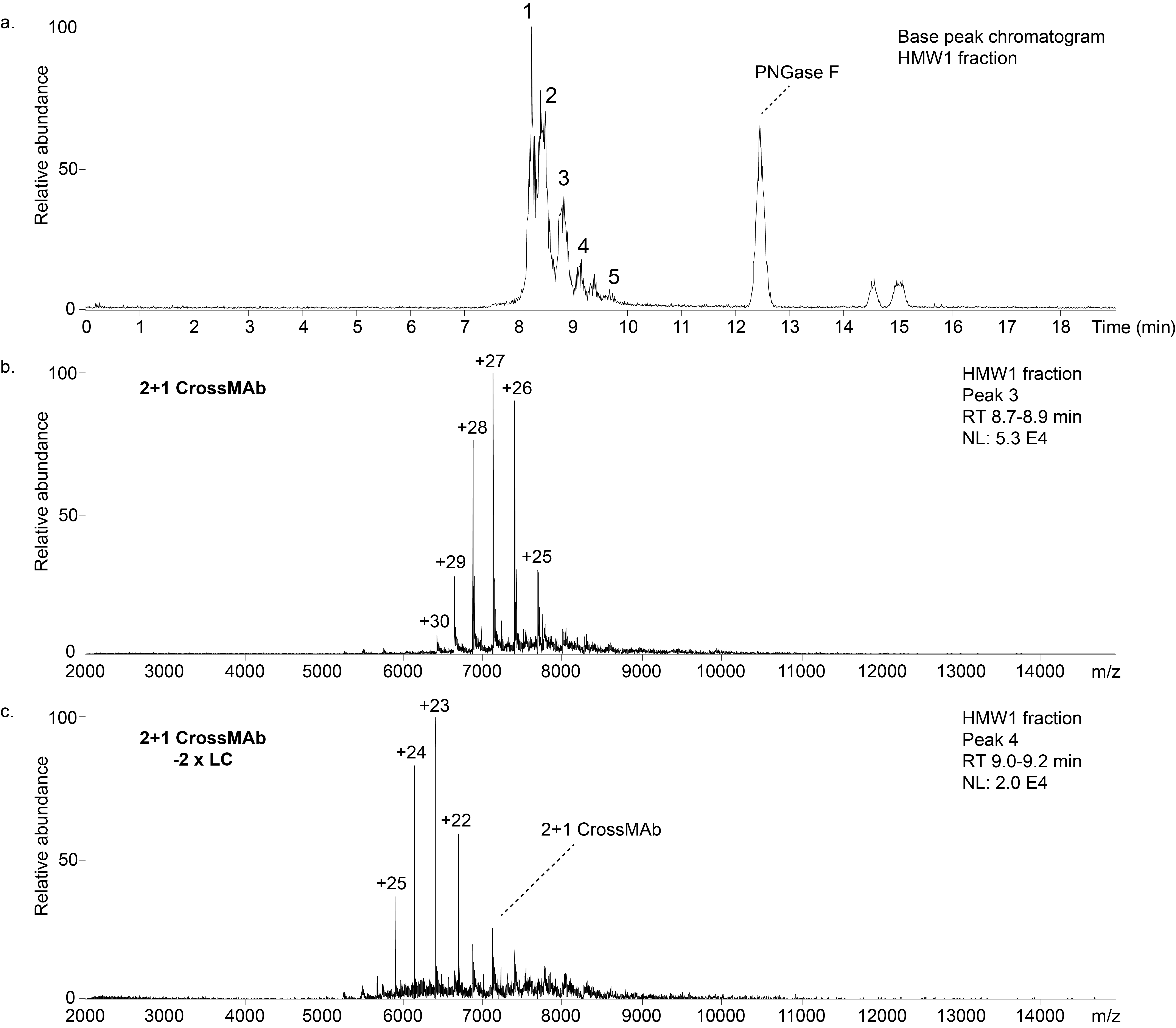


Supplementary Figure 8: **Other Species Present in The HMW1 Fraction.** a. Full base peak chromatogram of the HMW1 fraction from SEC-nMS analysis. Peaks are listed by numbers in ascending order and their native spectra are shown. Peak 1 and 2 are omitted as they appear in other figures. b. The 2+1 CrossMAb product is found at the same charge state distribution as previously observed. c. eluting directly after the 2+1 CrossMAb is the 2+1 CrossMAb which has lost either LC. Peak 5 is the knob half body at low abundance.


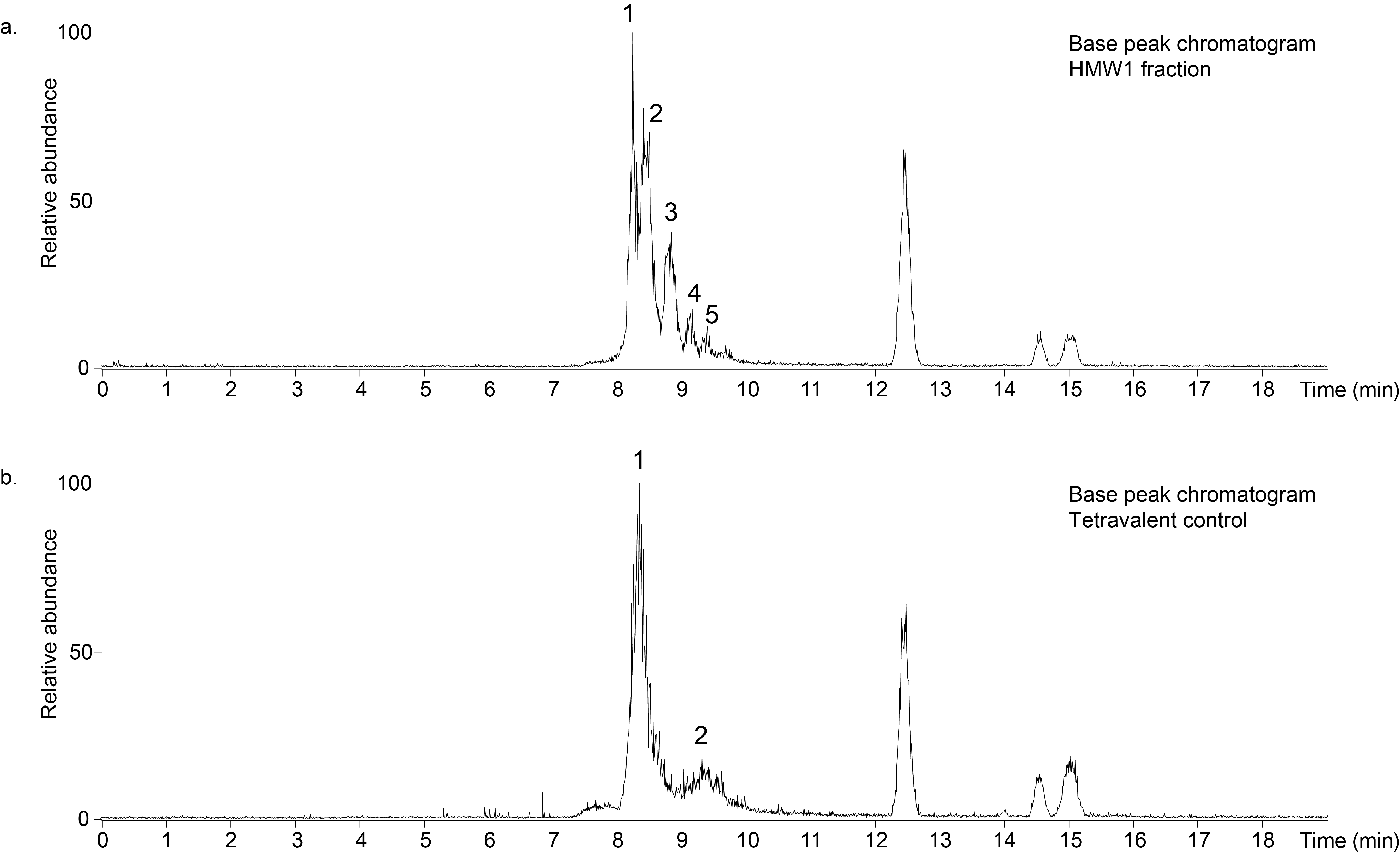


Supplementary Figure 9: **SEC Chromatogram Comparison of HMW1 and the Tetravalent Control.** The base peak chromatogram of the HMW1 fraction (a) compared to that of the tetravalent control (b) reveals that indeed only knob HCs are present, as the peaks corresponding to side products or fragments containing the 2+1 CrossMAb structure are no longer present. a. The HMW1 fraction shows 5 distinct peaks, whereas the tetravalent control (b) shows only 2 distinct peaks, the tetravalent variant and the knob half body.


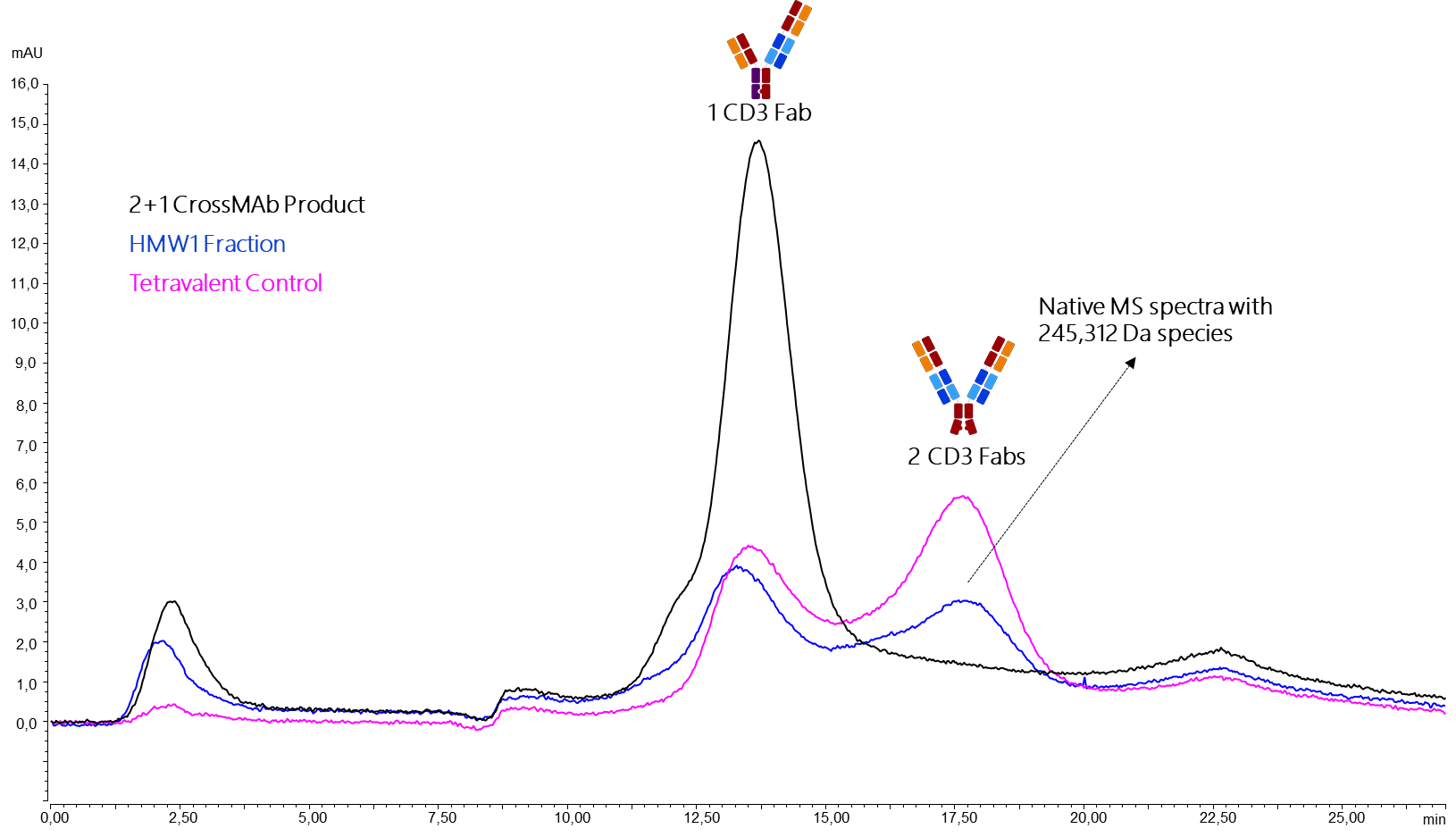


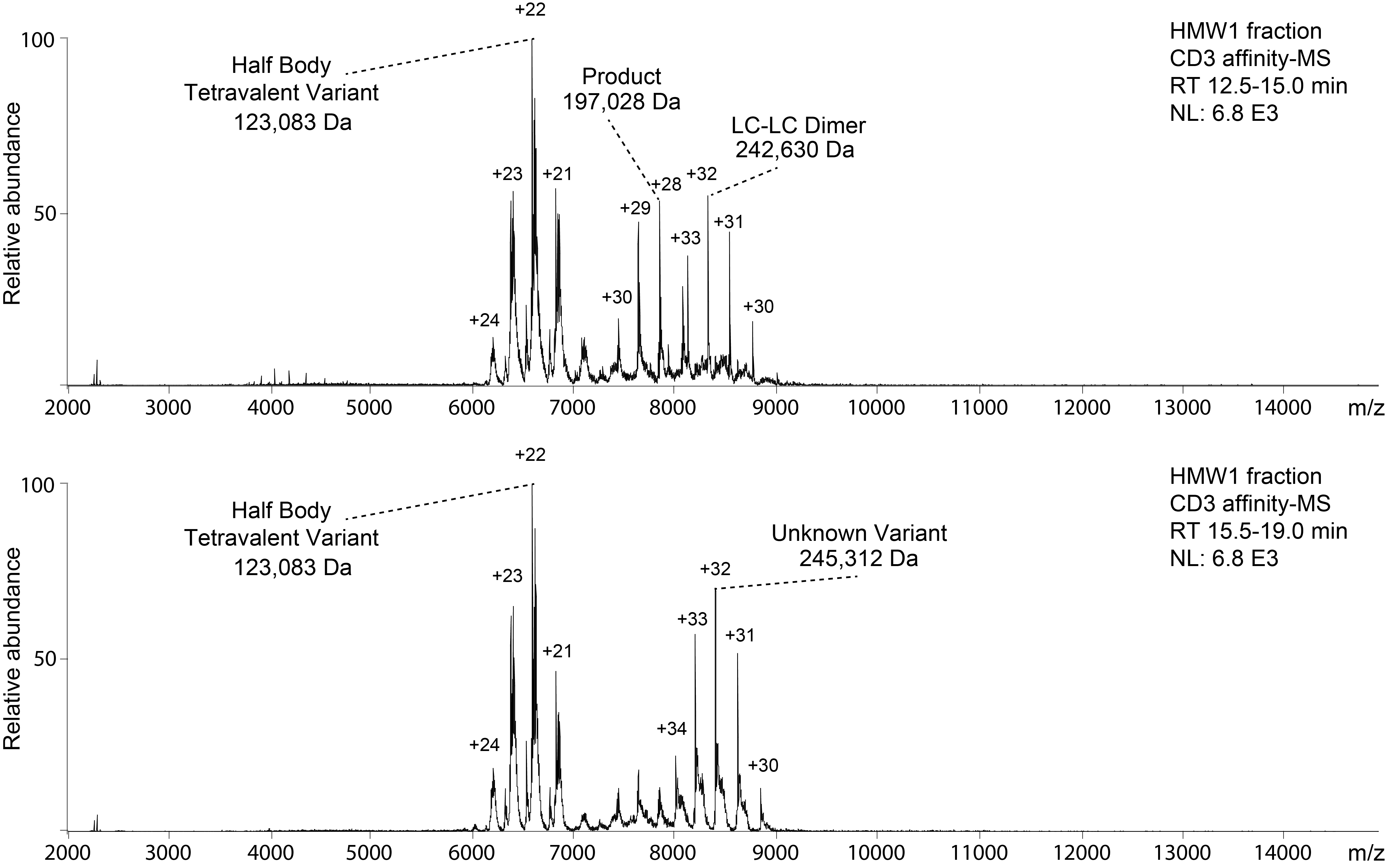


Supplementary Figure 10: **Native MS Using Online-Coupled CD3 Affinity Column**. By-products are separated by CD3 affinity based on the amount of CD3 Fabs they posses. As such, trivalent and tetravalent variants can be separated. A clear difference in RT is seen between the 2+1 CrossMAb and the tetravalent control. The HMW1 fraction eluting at the peak indicating tetravalent variants shows the mass of the unknown variant. Samples were not deglycosylated prior to analysis (the annotated mass is the mass of the unknown variant + 2×G0F glycans). Mass spectra with species annotations are provided for the HMW1 fraction. Mass spectra of the trivalent and tetravalent peak show the trivalent peak to contain the product and LC-LC dimer. The tetravalent peak shows the unknown variant as a tetravalent species. Half bodies of the non-covalent knob-knob variant elute or occur in both peaks due to bivalent retention and in source dissociation.


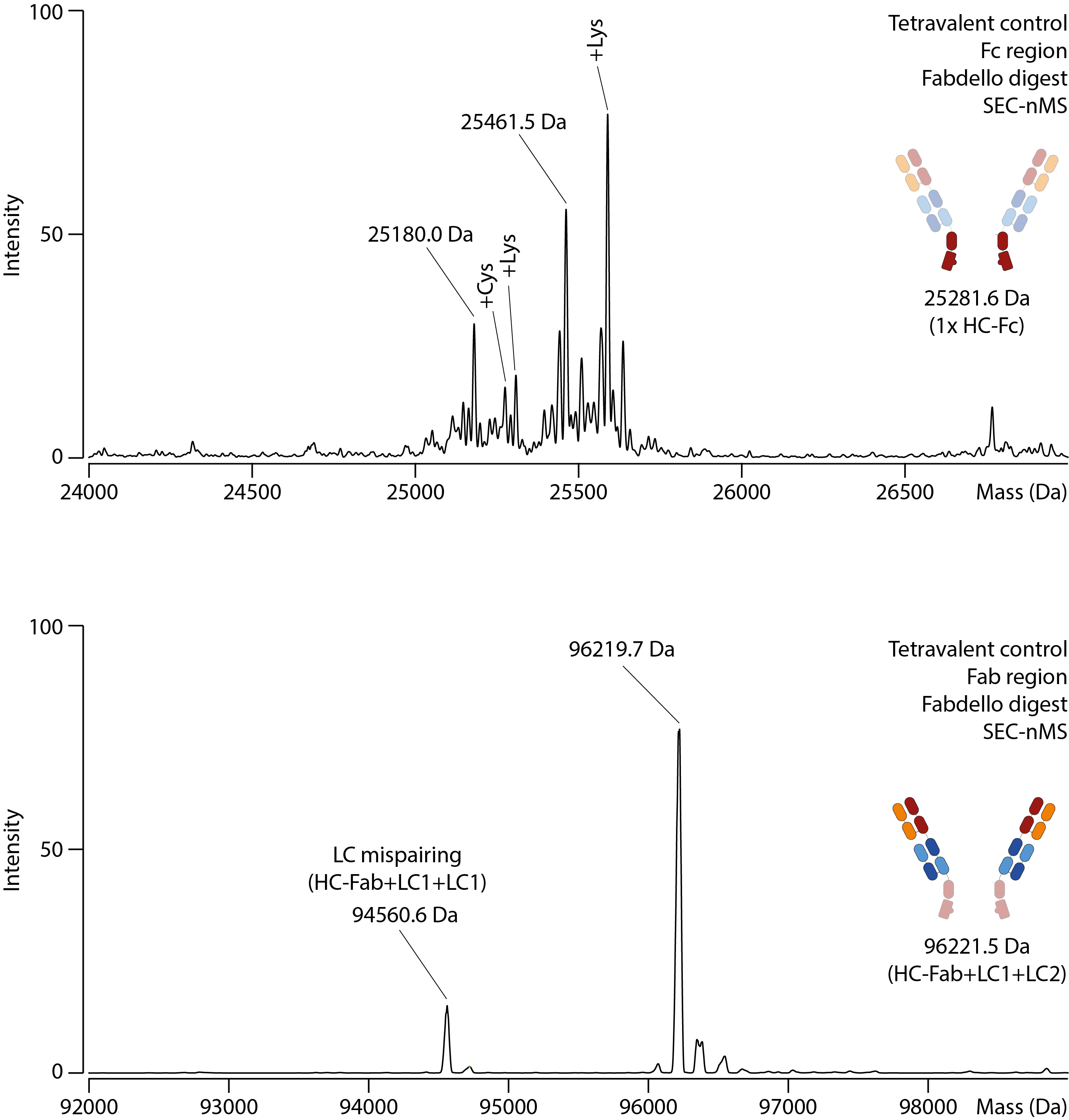


Supplementary Figure 11: **SEC-Nms After Fabdello Digest Localizes Heterogeneity on the Tetravalent Control.** Digestion with Fabdello prior to SEC-nMS allows analysis of the individual Fc and Fab parts of the tetravalent variant. This reveals the previously observed heterogeneity to be conserved only on the Fc part of the tetravalent variant. Additionally, we cannot annotate the mass of the knob HC Fc directly, meaning modifications have already taken place. For the knob Fc, we can see mass shifts corresponding to cysteinylation and additional lysines.


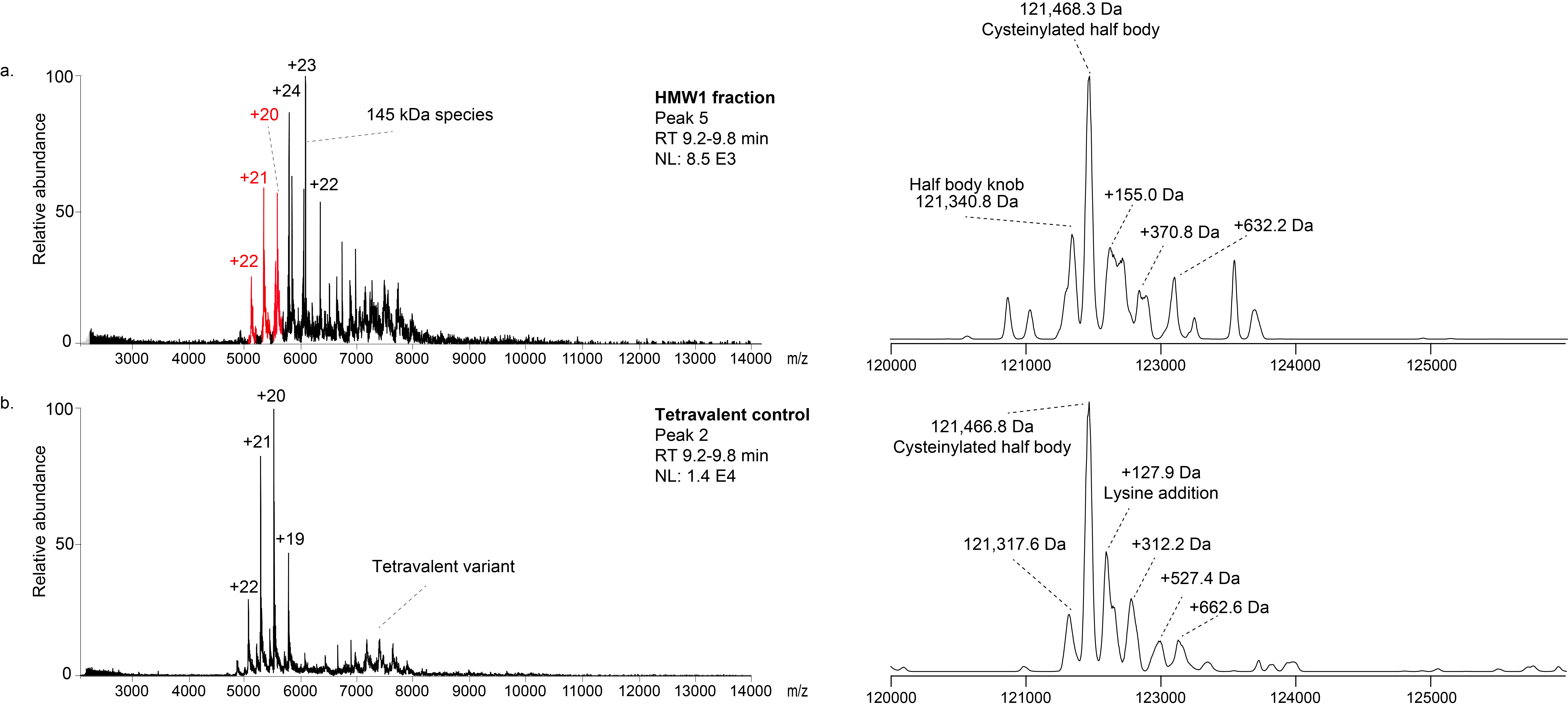


Supplementary Figure 12: **Comparison of Knob Halfbody Proteoforms between HMW1 and the Tetravalent Control on Native MS.** a. The raw SEC-nMS spectrum shows the co-elution of the knob half body of the HMW1 fraction (left) and the deconvoluted mass spectrum (right), which reveals its heterogeneity. b. In a similar fashion, the half-body of the rec2+2 control shows the same charge state distribution in the native spectrum (left) and a similar proteoform profile (right) as the HMW1 fraction knob half body.
